# Mechanism of Fatigue Induced by Different Cycling Paradigms With Equivalent Dosage

**DOI:** 10.3389/fphys.2020.00545

**Published:** 2020-05-29

**Authors:** Miao-Ju Hsu, Hsiao-Lung Chan, Ying-Zu Huang, Jau-Hong Lin, Heng-Hsiang Hsu, Ya-Ju Chang

**Affiliations:** ^1^Department of Physical Therapy, College of Health Science, Kaohsiung Medical University, Kaohsiung, Taiwan; ^2^Department of Physical Medicine and Rehabilitation and Department of Medical Research, Kaohsiung Medical University Hospital, Kaohsiung, Taiwan; ^3^Department of Electrical Engineering, College of Engineering, Chang Gung University, Taoyuan, Taiwan; ^4^Neuroscience Research Center, Chang Gung Memorial Hospital, Taoyuan, Taiwan; ^5^Department of Neurology, Chang Gung Memorial Hospital, Taoyuan, Taiwan; ^6^School of Medicine, College of Medicine, Chang Gung University, Taoyuan, Taiwan; ^7^School of Physical Therapy and Graduate Institute of Rehabilitation Science, College of Medicine, and Healthy Aging Research Center, Chang Gung University, Taoyuan, Taiwan

**Keywords:** exercise, central fatigue, peripheral fatigue, bicycling, maximal voluntary contraction

## Abstract

Leg cycling is one of the most common modes of exercise used in athletics and rehabilitation. This study used a novel cycling setting to elucidate the mechanisms, central vs. peripheral fatigue induced by different resistance with equivalent works (watt^∗^min). Twelve male adults received low and relatively high resistance cycling fatigue tests until exhausted (RPE > 18) in 2 weeks. The maximal voluntary contraction, voluntary activation level, and twitch forces were measured immediately before and after cycling to calculate General (GFI), central (CFI), and peripheral (PFI) fatigue indices of knee extensors, respectively. The results showed that the CFI (high: 92.26 ± 8.67%, low: 78.32 ± 11.77%, *p* = 0.004) and PFI (high: 73.76 ± 17.32%, low: 89.63 ± 11.01%, *p* < 0.017) were specific to the resistance of fatigue protocol. The GFI is influenced by the resistance of cycling to support the equivalent dosage. This study concluded that the mechanism of fatigue would be influenced by the resistance of fatigue protocol although the total works had been controlled.

## Introduction

Leg cycling is one of the most common modes of exercise used in athletics and rehabilitation to evaluate or promote cardiopulmonary endurance, increase lower extremity muscle strength and endurance, and maintain lower extremity range of motion (Kubukeli et al., 2002; Fang et al., 2015; Wei et al., 2018). The advantages of a stationary leg cycling exercise regimen include the ease of controlling exercise intensity and monitoring exercise responses. Leg cycling exercise interventions in individuals with motor dysfunction have shown that the effects of leg cycling exercise training can be translated into improved postural control in standing and better function during daily activities involving the lower extremities, such as walking speed and endurance (Katz-Leurer et al., 2006; Yang et al., 2014). It is also commonly used in athlete training for improving physical fitness (Jones et al., 2015; Paquette et al., 2017; Androulakis-Korakakis et al., 2018).

Fatigue plays a crucial role in limiting exercise performance. Traditionally, fatigue can be defined as any reduction in the maximal capacity to generate force or power output (Vollestad, 1997) or decreased performance (Edwards, 1981; Huang et al., 2017). During exercise at a constant power output, fatigue can be express as an increased sense of effort (Carroll et al., 2017). In sports, it is essential for understanding the mechanism of fatigue and developing an appropriate training strategy to overcome fatigue and improve the persistence of exercise. Researchers had put great efforts to elucidate the mechanisms and the sources of fatigue following exercise (Gandevia, 2001; Lepers et al., 2002; Amann et al., 2006, 2008; Boyas and Guevel, 2011; Millet, 2011; Elmer et al., 2013; Jubeau et al., 2014; Thomas et al., 2015, 2016; Tomazin et al., 2017; Ansdell et al., 2018).

Fatigue can be divided into central and peripheral components. Central fatigue is attributed to the processes within the central nervous system (CNS) that reduce neural drive to the exercising muscle and lead to a decrease in voluntary activation level (VA) and, subsequently, its performance (Taylor et al., 2016; Sidhu et al., 2018). Peripheral fatigue, i.e., muscle fatigue, is attributed to neuromuscular transmission, excitation-contraction coupling, or muscle bioenergetics (Gandevia, 2001; Chang et al., 2008; Boyas and Guevel, 2011; Chen et al., 2014; Huang et al., 2017). The proportion of central versus peripheral fatigue during a specific exercise is influenced by exercise mode as well as exercise intensity (Gandevia, 2001; Boyas and Guevel, 2011; Jubeau et al., 2014; Rossman et al., 2014; Thomas et al., 2015, 2016; Tomazin et al., 2017). Jubeau et al. (2014) suggested that the nature of fatigue depends on the type of exercise, such as a single-joint exercise versus a multiple-joint exercise. Rossman et al. (2014) compared fatigue after single-leg and double-leg knee extensor exhaustion exercise and found that the single-leg knee extensor exercise induced a greater degree of peripheral fatigue. Taylor et al. (2016) demonstrated that during isometric contractions, low but prolonged application of force tended to result in central fatigue, while short high-force contractions were predisposed to cause peripheral fatigue. Thomas et al. (2015) conducted a study to investigate peripheral and central fatigue in cyclists after 4-, 20, and 40 km timed trials and found that greater magnitude of peripheral muscle fatigue occurred after shorter time and higher intensity trial while more central muscle fatigue occurred after longer time and lower intensity trial. Additionally, Thomas et al. (2016) explored fatigue employing stationary cycling exercises at different constant-loads to exhaustion suggested that the extent of peripheral and central fatigue was exercise intensity-dependent.

Fatigability can be improved by exercise training (Zghal et al., 2015; Bachasson et al., 2016). Stationary cycling is a dynamic exercise involving at least two joints and has been widely used to enhance physical fitness in healthy as well as patient populations. Understanding the basic rules of resistance setting and physiological mechanisms of different components of fatigue, i.e., central vs. peripheral fatigue, would be important for setting a training program. However, the protocol used in the above mentioned studies might not be feasible in clinical setting wherein transferring patients from the ergometer to the force chair within a very short time might pose a difficulty or inconvenience. In addition, the resistances used in the testing protocols in previous studies were for athletes or healthy adults which might be too high for patients. According to Sargeant and Jones, 25% of maximum voluntary contraction recruited mainly type I fiber while 50% of maximum voluntary contraction recruited partial type II fibers (Sargeant and Jones, 2013). It is plausible to hypothesize that resistance within this range might be sufficed to induce resistance- specific fatigue phenomenon. Whether the resistance within a patient’s tolerable range, i.e., 25% vs. 50% max, could induce different types of fatigue is not clear. Therefore, we developed a customized force measurement system attached to a stationary cycling. In this, all measurements could be taken on the stationary cycling system concurrently, avoiding the trouble taken by patients to transfer between systems. We also aimed to compare the types of fatigue induced during stationary leg cycling exhaustion exercises at low versus relatively high resistance protocols.

## Materials and Methods

According to Hunter and Ansdell, females are much less fatigable than males (Ansdell et al., 2019), Female’s physical performance are also known to be negatively influenced by menstruation. In order to avoid bias caused by this gender factor, only male subjects were recruited. Twelve healthy male adults (mean ± SD age 22.83 ± 1.64 years) with a sedentary lifestyle or irregular exercise habits participated in two experimental sessions spaced 7 days apart (Table 1). This 7-day interval allowed full recovery of long lasting fatigue (Chang and Shields, 2002; Thomas et al., 2016). Exclusion criteria were: (1) skeletal muscular disease; (2) neuromuscular disease; (3) use of anti-depressant drugs; and (4) participating in lower limb strengthening exercise within a week before the study. This study was approved by the Institutional Review Board. Informed consent forms were obtained from all participants.

**TABLE 1:** Subject information.

Subject ID	Age (year)	Height (cm)	Weight (kg)	Domination (R/L)	Exercise habit
1	23	179	88	R	Upper limb weight training: 2 days per week
2	24	175	80	R	None
3	23	170	58	R	None
4	23	179	75	R	Core muscle and Pectoralis strength training: 3 days per week
5	23	168	63	R	Core muscle and Pectoralis strength training: 3 days per week
6	20	166	55	R	None
7	20	165	56	R	None
8	21	185	63	R	None
9	24	163	64	R	None
10	24	176	58	R	None
11	24	175	55	R	None
12	25	176	69	R	None
Mean	22.83	173.08	65.33		
*SD*	1.64	6.67	10.69		

**FIGURE 1:**
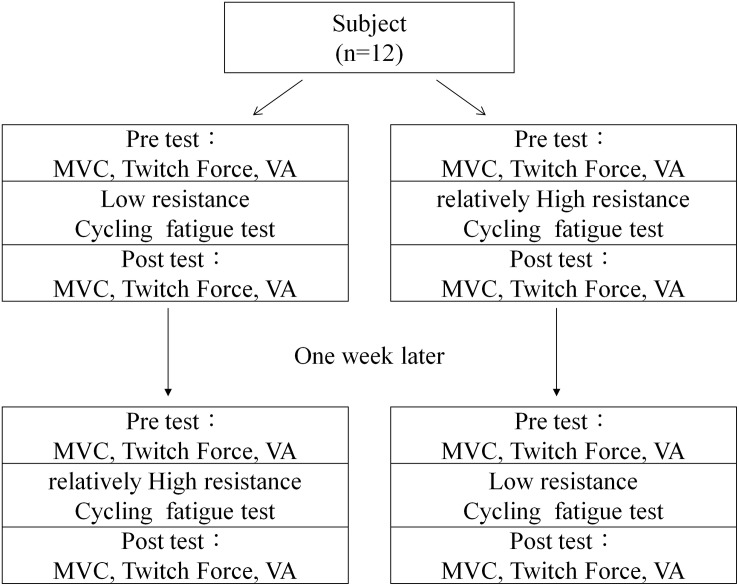
Flowchart of testing procedures.

After inclusion, demographic and anthropometric data of the subject were collected, such as height and weight. Subjects were then randomized into two groups according to a pre-randomized table. The first group received a low resistance cycling test in the first session and a relatively high resistance cycling test in the second session. The second group received the same two cycling tests in reverse order. The two test sessions had 1 week in between to allow the subject to fully recover from fatigue (Figure 1). All participants were blind to the purpose of the two protocols.

**FIGURE 2:**
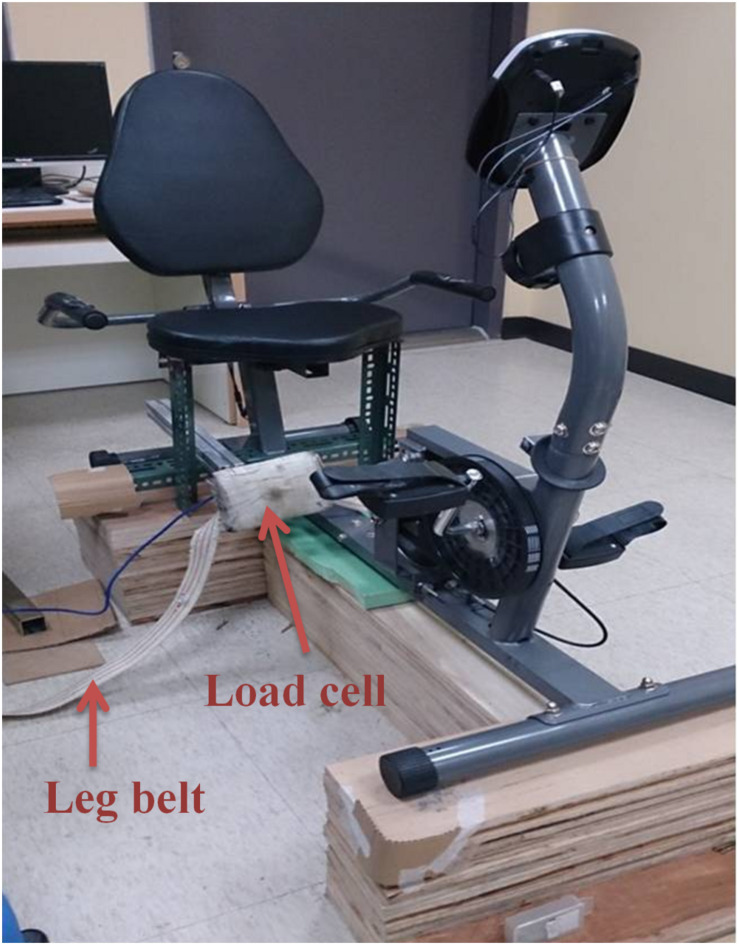
The cycling force measurement system (belts for fixing hips and trunk are not shown).

The cycling test utilized a customized force measurement system composed of a chair and a force transducer (Transcell Technology, IL, United States) to measure the knee extension force (Figure 2). During the exercise tests, the participant sat in the customized chair with their hip joint fixed at 90° flexion and their knee joint fixed at 90° flexion. The force transducer was fixed perpendicularly to the tibia bone and at 3 cm above the malleolus lines of ankle. The customized force measurement system was coupled with a stationary cycling system (Body Sculpture, United Kingdom) so the participant need not move position between tests. In order to evaluate the reliability of the system, the baseline values of the two experimental sessions were evaluated by intraclass correlation coefficient (ICC). Before each session, subjects were asked to casually pedal with no resistance to allow fine tune of the chair height and position of transducer to make sure no obstacles would be encountered during pedaling. After the setting was completed, subjects continued pedaling for about 1 min with no resistance as familiarization cycling.

Surface electrodes (9 × 12 cm) for electrical stimulation (stimulator model DS7A, Digitimer Ltd; Hertfordshire, United Kingdom) were placed on the muscle belly of the quadriceps, with one electrode approximately 4 cm above the superior border of the patella and the other electrode approximately 4 cm below the inguinal line. The positions of the electrodes were adjusted to produce only knee extension. Large electrodes were used because the quadriceps are large muscles and the current density delivered by large electrodes would be low and tolerable for subjects. An electrical pulse of 200 μs duration at supramaximal intensity was used for eliciting the twitch and interpolated twitch. The induced force was assessed simultaneously by a digital, real–time oscilloscope (TDS220, Tektronix Inc., United States), and the force signal was digitized using an analog-to-digital converter with 16-bit resolution (InstruNet Model 200 PCI controller, United States) at 1000 Hz. The stimulation intensity started low and progressively increased until no further increase in the twitch force was found. The intensity was then adjusted to 120% of the maximum intensity, which was defined as supramaximal intensity. This intensity was used for testing VA and twitch force which represented central and peripheral components of force (Chang et al., 2011; Huang et al., 2017; Chuang et al., 2019; Tang et al., 2020). To monitor the potential co-contraction, surface electrodes of an electromyographic recorder (B&L Engineering, CA, United States) were positioned on the hamstring muscle with the ground electrode placed on the patella for monitoring by an oscilloscope. If an obvious co-contraction of the hamstring occurred during any of the tests, the data were excluded and the test was repeated.

**FIGURE 3:**
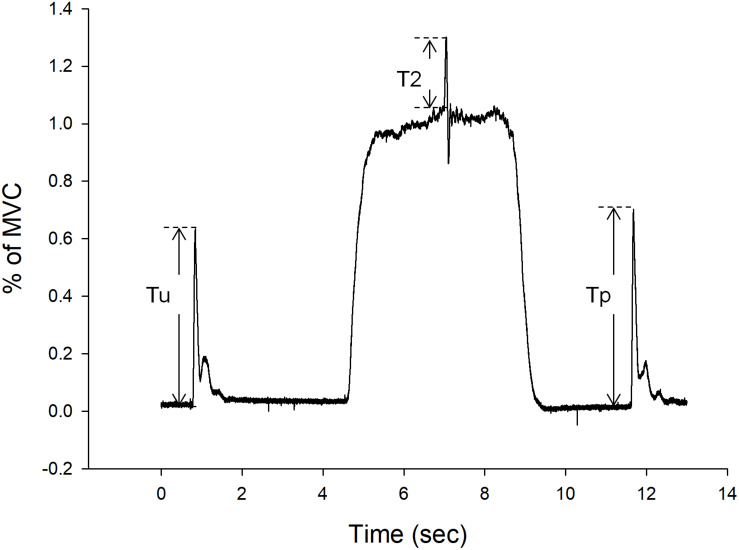
Representative force-time curves of voluntary activation (VA), and twitch force. During VA test, the Tu is the unpotentiated twitch and Tp is the potentiated twitch. Resting twitch force is the average of Tu and Tp. T2 is the interpolated twitch force.

Before each test session, participants performed three maximal voluntary contractions (MVCs) of the knee for practice and warm-up. After the warm-up, participants performed three more MVCs, each sustained for 5 s. After the MVC test, the VA of participants was evaluated by the interpolated twitch technique (ITT). The ITT is a non-invasive method for assessing the completeness of muscle activation during voluntary contractions, especially for a full muscular activation during a MVC (Huang et al., 2010; Chang et al., 2011). During this test, participants were asked to relax and then perform knee extensor MVCs. Maximal twitches were induced by supramaximal stimulation of the quadriceps during relaxation, at 2 out of 5 s during the MVC, and at 3 s after the MVC, to obtain the unpotentiated twitch, the interpolated twitch, and the potentiated twitch, respectively. The interpolated twitch represents the force generated from those motor units that failed to be activated by the CNS during MVC. In other words, the higher amount of interpolated twitch indicates a lower VA. This VA test was repeated two times with 5 s between repetitions. Representative data for VA, twitch force, and interpolated twitch force are shown in Figure 3. After the VA test, five supramaximal stimulations were delivered to the quadriceps muscle at 1 Hz to obtain the twitch forces to be analyzed.

At the beginning of the cycling test, subjects performed a 5-min warm-up by cycling at 60 revolutions per minute (RPM) with no resistance. After warming up, the cycling speed and resistance were adjusted according to the session (low or relatively high resistance) performed. The cycling speed increased by 3 RPM every 30 s until it reached 75 RPM for both relatively high and low resistance fatigue tests. The target resistance was set at half of that for the high resistance. According to the calibration, the target watt for low resistance cycling and high resistance cycling were 70 watt and 140 watt, respectively. The resistances corresponded to 25 and 50% MVC. Rate of perceived exertion (RPE) was reported by the participant every 1 min. Once the participant’s RPE reached 18 or the participant’s maximum heart rate (maximum = 220 minus the age), the cycling test was terminated and then 5 min of cool-down was provided, in which the cycling speed was reduced to 60 RPM and resistance was reduced gradually until the resistance reached 0. A 5-min cool down was added to comply with the exercise guideline for safety suggested by American College of Sports Medicine in order to prevent blood pooling in lower extremities and facilitate venous return (American College of Sports Medicine, 2016). After the cycling tests, MVCs, VA, and twitches were measured again to evaluate general fatigue, central fatigue, and peripheral fatigue.

The primary outcome variables include MVC, VA, and twitch force which represent the mixed, central, and peripheral components of force. The amplitude of MVCs and twitch forces were calculated from the force-time curve and represented as kg. The MVCs were the mean of 2 s after the peak value. The VA (ratio) was calculated from the formula 1, where T1 is the resting twitch force, which is the average of the unpotentiated and the potentiated twitches, and T2 is the interpolated twitch force (Figure 3).

(1) $$V\,A=\left(1-T_{2}/T_{1}\right)\times 100\%$$

A decrease in MVC, VA, and twitch force was considered to represent general fatigue, central fatigue, and peripheral fatigue, respectively. Therefore, the ratio of post-fatigue MVC to pre-fatigue MVC was calculated as general fatigue index (GFI). The ratio of post-fatigue VA to pre-fatigue VA was calculated as central fatigue index (CFI). The ratio of the post-fatigue twitch force to the pre-fatigue twitch force was calculated as peripheral fatigue index (PFI) (Chien et al., 2008; Ju et al., 2011). Higher fatigue index indicates less fatigue.

Two-way (time by resistance level) repeated measure of ANOVA was used to analyze the change in MVC, VA, and twitch force. One way repeated measure of ANOVA was used to analyze the GFI, CFI, and PFI difference after two resistance levels of cycling tests. The secondary outcome variables were the time to fatigue (RPE ≥ 18) and average RPE, which was calculated from the RPE values reported by the subject every minute. Paired *t*-test was used to analyze the difference between the average RPE values measured at the two resistance levels. The significance level was set at *p* < 0.05.

## Results

The baseline data before both low and relatively high resistance cycling tests are listed in Table 2. No statistically significant differences were found, suggesting that subjects were not influenced by the previous cycling test session. The ICC were high (>0.75) (Portney and Watkins, 2015) for MVC, VA, and twitch force (Table 3).

**TABLE 2:** The baseline data before both resistance cycling tests.

Item	Low resistance	High resistance	*P*
MVC (kg)	39.26 ± 9.63	38.05 ± 8.35	0.33
VA (%)	73.99 ± 10.64	74.08 ± 9.81	0.95
TW (kg)	18.84 ± 5.43	18.23 ± 3.46	0.61

*MVC, maximal voluntary contraction. VA, voluntary activation level. TW, twitch force.*

**TABLE 3:** The intraclass correlation coefficient (ICC) of maximal voluntary contraction, voluntary activation level, and twitch force.

	ICC
MVC	0.943696865
VA	0.943357353
TW	0.771961487

*MVC, maximal voluntary contraction. VA, voluntary activation level. TW, twitch force.*

**FIGURE 4:**
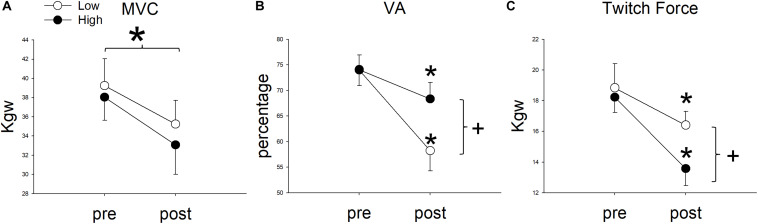
The comparison of **(A)** maximal voluntary contraction (MVC), **(B)** voluntary activation (VA), and **(C)** Twitch Force between pre and post fatigue in cycling exercises of two resistances. *Significant between pre and post fatigue in both resistance cycling exercises (*P* < 0.05). +Significant between low and relatively high resistance cycling exercise (*P* < 0.05).

**FIGURE 5:**
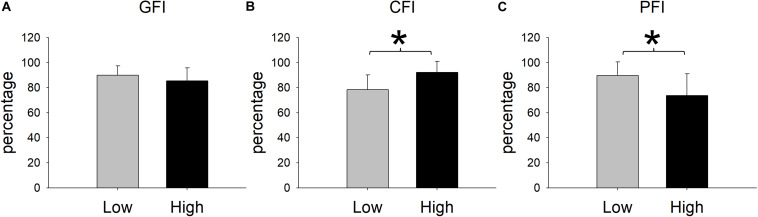
Cycling-induced fatigue indices in the two resistances. CFI represents central fatigue index; PFI represents peripheral fatigue index; and GFI represents general fatigue index. *Significant between low and relatively high resistance cycling exercise (*P* < 0.05).

Upon reaching fatigue at low resistance, the MVC decreased from 39.26 ± 9.63 kg to 35.24 ± 8.61 kg. Upon reaching fatigue at relatively high resistance, the MVC decreased from 38.05 ± 8.35 kg to 33.07 ± 10.58 kg (Figure 4A). The interaction between resistance level and time (pre- versus post-fatigue) was not significant [*F*(1,11) = 0.34, *p* = 0.571], but the effect of fatigue on MVC was significant [*F*(1,11) = 76.42, *p* < 0.001]. This suggested that both low and relatively high resistance cycling decreased MVC to a similar extent. The GFI was 89.95 ± 7.33% in the low resistance cycling test and 85.45 ± 10.32% in the relatively high resistance test (Figure 5A). The difference was not significant [*F*(1,11) = 1.45, *p* = 0.253], suggesting that low resistance and relatively high resistance cycling caused similar amounts of general fatigue.

In terms of VA, the repeated measures ANOVA showed that the interaction between cycling resistance and time was significant [*F*(1,11) = 14.08, *p* = 0.003], suggesting that the resistance level had different effects on the VA. The VA decreased from 73.99 ± 10.64% to 58.24 ± 13.79% with low resistance (*p* < 0.001), whereas with relatively high resistance, it decreased from 74.08 ± 9.81% to 68.34 ± 11.19% (*p* = 0.008; Figure 4B). The amount of decrease in the VA was larger after low resistance cycling than after relatively high resistance cycling. The CFI was 78.32 ± 11.77% at low resistance and 92.26 ± 8.67% at relatively high resistance (Figure 5B). This difference was significant [*F*(1,11) = 12.98, *p* = 0.004], suggesting that low resistance cycling caused more central fatigue than relatively high resistance cycling.

In terms of twitch force, the repeated measures ANOVA showed that the interaction between cycling resistance and time was significant [*F*(1,11) = 5.75, *p* = 0.035], suggesting that the level of resistance had different effects on the twitch force. After fatigue was reached at low resistance, the twitch force decreased from 18.84 ± 5.43 kg to 16.41 ± 3.09 kg (*p* = 0.0124), whereas after relatively high resistance fatigue, the twitch force decreased from 18.23 ± 3.46 kg to 13.58 ± 3.87 kg (*p* < 0.001; Figure 4C). The decrease in twitch force was larger after relatively high resistance cycling than after low resistance cycling. The PFI was 89.63 ± 11.01% in the low resistance cycling test and 73.76 ± 17.32% in the high resistance test (Figure 5C). A significant difference was observed [*F*(1,11) = 7.84, *p* = 0.017], suggesting that relatively high resistance cycling caused more peripheral fatigue than low resistance cycling.

The time to fatigue (RPE ≥ 18, “extremely hard”) for low resistance cycling (26.92 ± 5.93 min) was significantly longer than that for relatively high resistance cycling [11.58 ± 5.92 min; *F*(1,11) = 86.53, *p* < 0.001]. The estimated works for low and high resistance cycling were 1884.17 ± 415.16 and 1621.67 ± 828.16 watt^∗^min [*F*(1,11) = 1.69, *p* = 0.221], respectively. The average minute by minute RPE during the low resistance cycling test was 8.63, which is equivalent to “very light.” The average minute by minute RPE during the relatively high resistance cycling test was 12.5, which is equivalent to “somewhat hard.” This result confirmed that the average exertion perceived by participants was different for the two resistance levels.

## Discussion

Our study used a novel cycling system to evaluate the influence of resistance on fatigue and found that the mechanism of fatigue is resistance dependent. The low resistance cycling induced mainly central fatigue whereas the relatively high resistance cycling induced mainly peripheral fatigue. Our study also showed that using RPE as stopping criteria (RPE ≥ 18), both relatively high and low resistance paradigm induced similar amount of general fatigue and produce similar works (watt^∗^min) via different (long duration for low resistance and short duration for high resistance) duration. This study is the first study to show the task specific fatigue in lower intensity cycling exercise protocols and is the first study to develop a cycling test of fatigue requiring no weight transferring of subjects.

Previous studies supported that the relative contribution of central and peripheral fatigue depends upon the duration and intensity of exercise (Burnley et al., 2012; Thomas et al., 2015) in that peripheral fatigue contributes relatively more to MVC reduction after short, high-intensity exercise, and central fatigue contributes relatively more during longer-duration, moderate intensity exercise (Carroll et al., 2017). A similar response in cycling exercise was also found in our study. Thomas et al. tested twelve well-trained male cyclists and found that fatigue is task specific which is dependent on intensity and duration domains (Thomas et al., 2016). Our study supported that the task specific fatigue also holds true in low intensity. The exercise intensity used in our study was much lower than that used in Thomas et al.’s (2016) study (140 watt vs. 399 watt). Based on the RPE data, the averaged exercise intensities elicited by our cycling protocols were very light and moderate for our low resistance (initial RPE = 8.63) and relatively high resistance protocols (initial RPE = 12.50), respectively. This finding is particularly important for the training of more frail population, such as elder and patient populations due to their low tolerance of exercise. Differentiate the components fatigue is important for these populations. According to previous studies, individuals with multiple sclerosis and individuals with Parkinson disease prone to have central origin force loss and central fatigue (Chang et al., 2011; Huang et al., 2017). The peripheral origin force loss was crucial for early age-related weakness (Chuang et al., 2019). In Thomas et al.’s study, the post fatigue tests were done within 2 min post exercise. This time period was too short for elder and patient populations to transfer between bike and testing chairs. Our study may provide a feasible paradigm to differentiate the origin of weakness and fatigue during the progress of disease and training for elder and patient populations.

Other researchers used isometric model to study the influence of resistance on the mechanism of fatigue. Boyas et al. (2013) employed sustained isometric plantarflexor contraction at 25, 50, and 75% of maximal isometric torque and found that compared to the 25% exercise, the 50 and 75% exercises involved mostly peripheral fatigue. Though the form of exercise is different, our results are consistent with Boyas’s study. In our study, the low and relatively high resistances used in our study corresponds to 25 and 50% MVC. These two intensities also produced predominant central and peripheral fatigue, respectively. This suggests that the task specific fatigue is not limited to the forms of exercise, e.g., dynamic or isometric exercises.

Regarding the dosage equivalence issue, the dose equivalence could be evident in three aspects, i.e., RPE, total work, and GFI. The dose equivalence comparison is important and past researchers had done great efforts to achieve this. For example, in Thomas et al.’s (2016) study, they controlled the dose based on their pilot study to estimate the exercise duration for different cycling intensities. Their study showed the final RPE was similar in different intensities (Thomas et al., 2016). In our study, we controlled only the final RPEs (≥18) for both low and relatively high resistance cycling. The duration to exhaustion was not controlled but were varied for high and low resistance cycling exercises and made the resultant works calculated by watt^∗^min to be similar (*p* > 0.05). Similarly, Rannou et al.’s (2019) controlled the RPEs achieved during one-legged knee extension exercise at different intensities to exhaustion (85-5%, 85%, and 85 + 5% peak power output). The GFIs were also similar after both low and relatively high resistance cycling exercises. These results suggested that RPE is capable of quantifying the total dosage, exercise works and general fatigue, of exercise. However, the different distributions of central and peripheral fatigue would occur under the same amount of effort (or total dosage) and thus should be quantified in clinical and/or athlete trainings.

There are several possible mechanisms for low resistance cycling to induce more central fatigue. One possible mechanism is related to the group III/IV afferents (Allen et al., 2010). During cycling, contraction-induced mechanical and chemical stimuli activate III/IV muscle afferents and raise the spontaneous discharges (Kaufman et al., 2002). These sensory neurons project to various sites within the CNS such as motor cortex, insular, and cingulate cortex (Klass et al., 2008). Strong feedback from these muscle afferents limited voluntary descending drive from the motor cortex and restricted motoneuronal output and thus decreased muscle activation level (Sidhu et al., 2014). Researchers also suggested that group III/IV muscle afferents primarily dysfacilitate spinal motoneurons and facilitate motor cortical cells during non-fatiguing cycling exercise. In contrast, when fatigue occurs, group III/IV muscle afferents primarily dysfacilitate/inhibit the motor cortex (Sidhu et al., 2017). The results of our study further suggested that using a less challenging exercise (low resistance) to the level of exhaustion, more central fatigue would be developed, compared to a more challenging exercise (relatively high resistance) to the same exhaustion level. It is possible that, in low resistance, there is enough time for group III/IV to dysfacilitate the motor cortex and produce central fatigue before the peripheral fatigue. In contrast, in relatively high resistance, the peripheral fatigue developed before motor cortex dysfacilitation.

Central fatigue has also been suggested to be related to neurotransmitters such as serotonin and dopamine (Meeusen et al., 2006; Tong et al., 2015). In animal studies, dopamine has been shown to increase during exhausting exercise (Hu et al., 2015), and a reduced level of dopamine was reported in fatigued rats (Roelands and Meeusen, 2010). The dopaminergic system and the serotonin pathway could produce limbic modification of cortical motor output (Chaudhuri and Behan, 2000) and thus affect motivation and influence the ability to sustain VA of muscle. In our study, the subjects are healthy individuals. The different central fatigue responses in two resistance cycling paradigms might not be explained by the deficits of dopaminergic or serotonergic system deficits. It is possible that low resistance cycling would result in more challenges to the brain neurotransmissions than relatively high resistance cycling to the similar exhausted level.

In contrast, our study showed that the relative high resistance cycling exercise induced peripheral fatigue before the CNS limiting the voluntary output. The sources of peripheral fatigue include low frequency fatigue (LFF) and high frequency fatigue (HFF). LFF usually causes a prolonged low-frequency force depression and is associated with a failure in the excitation–contraction coupling; intracellular measurements have shown that LFF is due to reduced Ca^2+^ release (Westerblad and Allen, 1993; Shields and Chang, 1997; Shields et al., 1998; Hill et al., 2001; Allen et al., 2008), decreased myofibrillar Ca^2+^ sensitivity (Bruton et al., 2008), and reduced level of proteins involved in transverse (T)-tubule and sarcoplasmic reticulum membrane apposition (Corona et al., 2010). HFF usually has a rapid recovery and is attributed to an accumulation of extra-cellular K+. For this type of fatigue, rapid recovery of force occurs when the frequency is reduced (Jones, 1996; Shields and Chang, 1997; Chang and Shields, 2002; Shields et al., 2006; Millet et al., 2011). In our current study design, we could not quantify the proportion of HFF and LFF. However, both fatigue could contribute to certain extent since our post-fatigue test was performed immediately post cycling.

The VA at baseline in our study was approximately 75%, which is comparable to but slightly less than that reported in previous studies for healthy individuals (Colson et al., 2009). For example, Colson et al. (2009) reported 81.45–85.89% VA. This could not be attributed to subjects’ familiarization. Before formal testing, subjects usually have several warm-up contractions. The interpolated twitch technique has proved to be valid and used in testing knee extensors of healthy (Huang et al., 2010), Parkinson’s disease (Huang et al., 2017), multiple sclerosis (Chang et al., 2011), obstructive sleep apnea (Chien et al., 2010), and ACL injured individuals (Tang et al., 2020). The high ICC of VA also supported that the reliability was high (Table 3). One contributing factor to the inter-study differences may be the angles of the knee joints, which were 70° and 90° for Colson’s and ours, respectively. Additionally, the voluntary activation level is associated with the physical activity of individuals (Bogdanis, 2012). Over 50% of our subjects were sedentary, defined as exercising less than one time per week and thus were likely less accustomed to fully activate motor units. Another potential difference was that Colson et al. (2009) used nerve stimulation whereas we used surface pad stimulation. The vastus intermedius was hard to be activated through surface stimulation and might contribute to the lower VA.

The results of this study will have several practical applications. The two paradigms used in this study could serve as a model to induce different type of fatigue and discriminate individuals’ weighting of central and peripheral fatigue for future studies while developing new therapeutic treatments for fatigue. This study could help in developing cycling paradigm for training different types of athletes, such as central vs. peripheral strength domain sports. In addition, in our current testing setting the force transducer is mounted on ergometer which does not require the transfer of subjects and thus provides the possibility to quantify fatigue during cycling.

There are some limitations to bear in mind when interpreting the results of this study. First, the subjects recruited in the present study were generally healthy but sedentary male individuals. For female individuals, future study is suggested for testing with control in menstruation factors. Second, the post-fatigue measurements were taken after the cool-down period which might involve some level of recovery. This might underestimate some amount of central fatigue but not peripheral fatigue. According to previous studies of animal and human models, the recovery of peripheral fatigue was still obvious up to 30 min post fatigue protocol (Place et al., 2004; Ross et al., 2007, 2010; Sidhu et al., 2009; Temesi et al., 2014).

## Conclusion

This study concluded that the mechanism of fatigue would be influenced by the nature of exercise protocol. Both low and relatively high resistance leg cycling can induce fatigue. The level of resistance during leg cycling influences the proportion of central and peripheral fatigue. Low resistance cycling appears to cause more central fatigue than relatively high resistance exercise. Quantifying GFI might not be sufficient for clinical training.

## Data Availability Statement

The datasets generated for this study are available on request to the corresponding author.

## Ethics Statement

The studies involving human participants were reviewed and approved by the Institutional Review Board of Chang Gung Memorial Hospital. The patients/participants provided their written informed consent to participate in this study.

## Author Contributions

M-JH, H-LC, Y-ZH, J-HL, H-HH, and Y-JC conceived, designed, and coordinated the study. M-JH, H-HH, and Y-JC acquired the data. M-JH, H-LC, and Y-JC analyzed and interpreted the data, and were a major contributor in writing the manuscript. All authors read and approved the final manuscript.

## Conflict of Interest

The authors declare that the research was conducted in the absence of any commercial or financial relationships that could be construed as a potential conflict of interest.
